# Regulation of tumor growth by circulating full-length chromogranin A

**DOI:** 10.18632/oncotarget.12237

**Published:** 2016-09-24

**Authors:** Flavio Curnis, Alice Dallatomasina, Mimma Bianco, Anna Gasparri, Angelina Sacchi, Barbara Colombo, Martina Fiocchi, Laura Perani, Massimo Venturini, Carlo Tacchetti, Suvajit Sen, Ricardo Borges, Eleonora Dondossola, Antonio Esposito, Sushil K. Mahata, Angelo Corti

**Affiliations:** ^1^ Division of Experimental Oncology, IRCCS San Raffaele Scientific Institute, Milan, Italy; ^2^ Experimental Imaging Center, IRCCS San Raffaele Scientific Institute, Milan, Italy; ^3^ University of California, Los Angeles, CA, USA; ^4^ La Laguna University, Tenerife, Spain; ^5^ Vita-Salute San Raffaele University, Milan, Italy; ^6^ VA San Diego Healthcare System and University of California, San Diego, La Jolla, CA, USA

**Keywords:** chromogranin A, angiogenesis, tumor perfusion, endothelial cells, protease-nexin-1

## Abstract

Chromogranin A (CgA), a neuroendocrine secretory protein, and its fragments are present in variable amounts in the blood of normal subjects and cancer patients. We investigated whether circulating CgA has a regulatory function in tumor biology and progression. Systemic administration of full-length CgA, but not of fragments lacking the C-terminal region, could reduce tumor growth in murine models of fibrosarcoma, mammary adenocarcinoma, Lewis lung carcinoma, and primary and metastatic melanoma, with U-shaped dose-response curves. Tumor growth inhibition was associated with reduction of microvessel density and blood flow in neoplastic tissues. Neutralization of endogenous CgA with antibodies against its C-terminal region (residues 410-439) promoted tumor growth. Structure-function studies showed that the C-terminal region of CgA contains a bioactive site and that cleavage of this region causes a marked loss of anti-angiogenic and anti-tumor potency. Mechanistic studies showed that full-length CgA could induce, with a U-shaped dose-response curve, the production of protease nexin-1 in endothelial cells, a serine protease inhibitor endowed of anti-angiogenic activity. Gene silencing or neutralization of protease nexin-1 with specific antibodies abolished both anti-angiogenic and anti-tumor effects of CgA. These results suggest that circulating full-length CgA is an important inhibitor of angiogenesis and tumor growth, and that cleavage of its C-terminal region markedly reduces its activity. Pathophysiological changes in CgA blood levels and/or its fragmentation might regulate disease progression in cancer patients.

## INTRODUCTION

Human chromogranin A (CgA) is a 439 residue-long protein stored in the dense-core granules of many neuroendocrine cells and neurons, and exocytotically released into the blood to reach about 0.5 nM levels [1, 2]. CgA is also expressed in lower amounts by granulocytes, cardiomyocytes and, in certain conditions, by keratinocyte [1, 3-5]. An increasing number of studies have shown that CgA and certain CgA-derived peptides, called vasostatin-1, pancreastatin, catestatin and serpinin, can regulate the cardiovascular system and metabolism, suggesting that this protein can exert extra-cellular functions [1]. Increased levels of CgA have been immunologically detected in the blood of patients with neuroendocrine tumors or with tumors that undergo neuroendocrine differentiation, including prostate, breast and non-small cell lung cancer [2, 6-8]. For these reasons CgA is widely used as a serological marker for neuroendocrine tumor diagnosis or for monitoring tumor progression/regression after therapy. However, patients with non-neuroendocrine tumors may also have abnormal levels of circulating CgA. For example, increased levels of immunoreactive CgA have been observed in a subpopulation of patients with non-small cell lung cancer lacking neuroendocrine cells in tumor tissues and in cancer patients treated with proton pump inhibitors, a class of drugs commonly used to treat acid peptic disorders [8-11]. Furthermore, elevated serum levels of CgA have been observed in patients with renal failure, heart failure, hypertension, rheumatoid arthritis, atrophic gastritis, inflammatory bowel disease, sepsis and other inflammatory diseases [1, 2, 8, 12-23]. Using specific assays, we recently observed that CgA is present in the blood of normal subjects and cancer patients as a mixture of full-length protein and fragments, including the N-terminal fragment CgA_1-76_ (vasostatin-1) and other fragments lacking part or the entire C-terminal region [24, 25]. Interestingly, the plasma levels of the fragment CgA_1-373,_ which is not or minimally present in normal subjects, is increased in the peripheral blood (and even more in the bone marrow plasma) of patients with multiple myeloma, while full-length CgA is decreased, pointing to increased CgA C-terminal fragmentation in these patients [25]. Thus, variable levels of full-length CgA and fragments are present in the blood of cancer patients for a variety of reasons.

A central question that we have yet to answer is whether the neuroendocrine release of CgA in circulation and its fragmentation can affect the biology and progression of non-neuroendocrine tumors.

Interestingly, recent studies have shown that CgA_1-439_ and the N-terminal fragment CgA_1-76_ inhibit angiogenesis, whereas the fragment CgA_1-373_ stimulates angiogenesis in various angiogenesis assays [24, 26]. Furthermore, full-length CgA_1-439_ and CgA_1-78_ can protect the endothelial barrier from the disassembly of vascular endothelial-cadherin adherence junctions, gap formation, and vascular leakage [27, 28], and reduce the traffic of tumor cells through the endothelium [29]. Although these findings suggest that CgA and some of its fragments might affect tumor biology, the pathophysiological relevance of circulating full-length CgA and the potential impact of CgA fragmentation on tumor progression remains to be established.

These notions prompted us to explore the potential role of circulating CgA as a regulator of tumor growth. As vascularized tumors are exposed to CgA released in circulation by the neuroendocrine system, we decided to perform the study using various murine models of non-neuroendocrine solid tumors, including subcutaneous fibrosarcomas, Lewis lung carcinoma and mammary adenocarcinomas, as well as orthotopic subcutaneous and metastatic (lung) melanomas.

We show that full-length CgA, but not fragments lacking the C-terminal region, can inhibit tumor growth in all these models, with U-shaped dose-response curves, and we provide evidence that an active site is located in the C-terminal region. Furthermore, we show that full-length CgA can impair angiogenesis, tumor perfusion and tumor growth through mechanisms depending on the induction of protease nexin-1, a serine protease inhibitor endowed of anti-angiogenic activity.

## RESULTS

### Exogenous CgA exerts anti-tumor effects with U-shaped dose-response curves in various murine models

**Figure 1:**
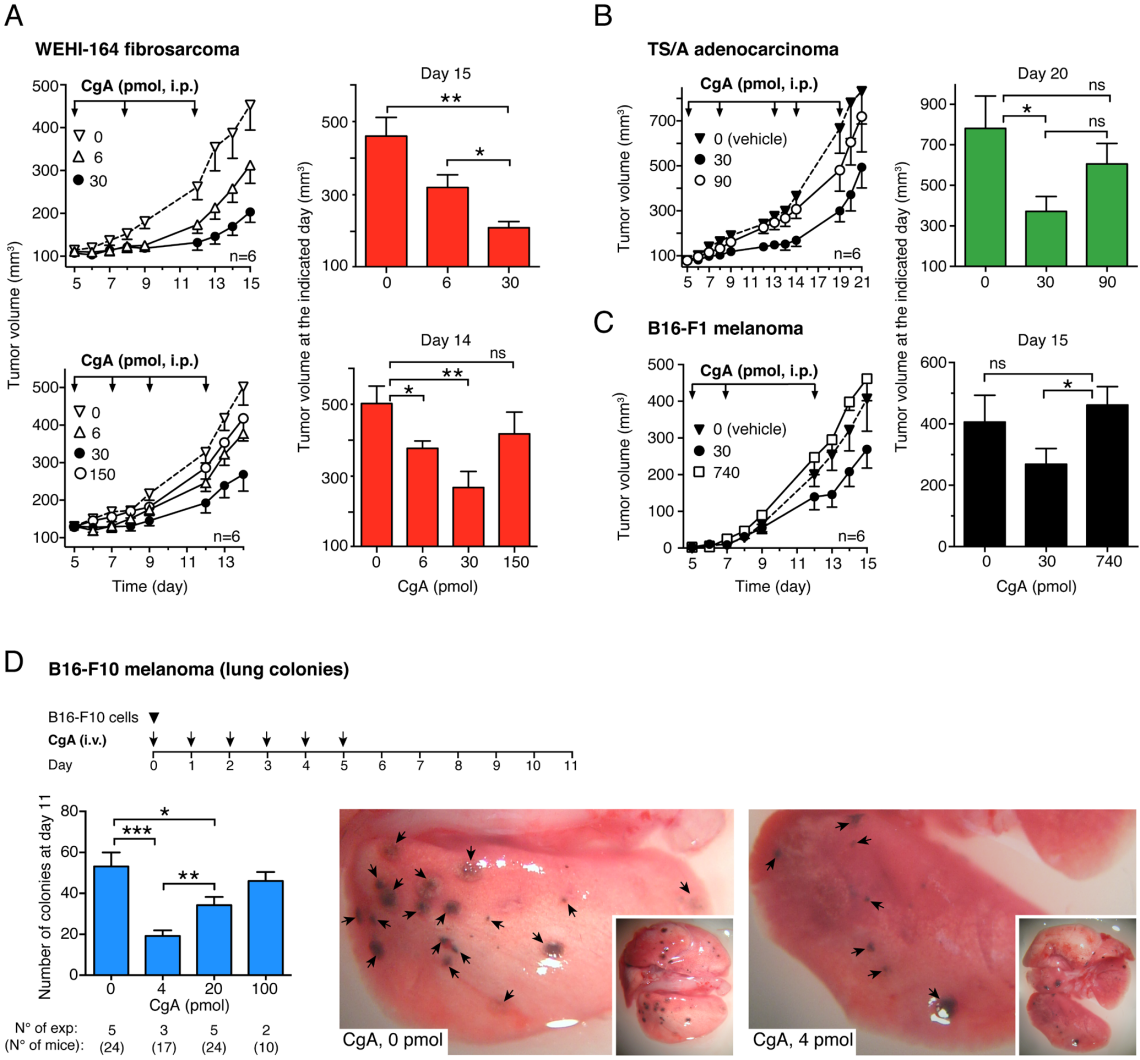
Effects of CgA on tumor growth in the WEHI-164 fibrosarcoma, TS/A adenocarcinoma and B16 melanoma models **A.***-***C.** *Effects of full-length CgA on the growth of subcutaneous tumors*. Tumor-bearing mice were treated (i.p.) at the indicated time (*arrows*) after tumor implantation with the indicated doses of recombinant CgA. Tumor volumes are shown (mean±SE, *n* = 6 mice/group). *, *P* < 0.05; **, *P* < 0.01, by *t* test (2-tailed).**D.** *Effects of CgA on lung colony formation by circulating melanoma cells*. C57BL/6 mice were injected with 6×10^4^ B16-F10 tumor cells (i.v.) and with recombinant CgA (i.v.) at the indicated time (arrows). At day 11, mice were sacrificed and the number of B16-F10 colonies in the lungs of mice was counted with the help of a stereomicroscope. Bars represent the number of colonies (mean±SE; number of mice/group reported below the graph). Representative photographs of lungs of mice treated with 0 and 4 pmol of CgA are also shown. *, *P* < 0.05; **, *P* < 0.01, and ***, *P* < 0.001 by *t* test (2-tailed).

The effect of exogenous recombinant and natural CgA (produced in *E.coli* cells and purified from pheochromocytomas, respectively) on tumor growth was investigated in different murine models, including WEHI-164 fibrosarcoma, TSA adenocarcinoma B16-F1 melanoma, and Lewis lung carcinoma (LLC), implanted subcutaneously in syngeneic mice. Furthermore, a metastasis model based on lung colonization by B16-F10 melanoma cells injected intravenously in mice was also used. Administration of 6 to 30 pmol of human recombinant full-length CgA to WEHI-164-bearing mice (i.p., every 3-4 days, three times) significantly delayed the growth of subcutaneous tumors in a dose-dependent manner (Figure 1A, upper panels). However, increasing the dose to 150 pmol resulted, paradoxically, in a lower effect (Figure 1A, lower panels). U-shaped dose response curves were also observed in the TS/A and B16-F1 subcutaneous models, after treatment (i.p.) with 0, 30, 90 or 0, 30, 740 pmol of recombinant CgA, respectively (Figure 1B and 1C) as well as in the B16-F10 lung-colonization model, after treatment (i.v.) with 0, 4, 20 and 100 pmol of CgA (Figure 1D). Of note, the 740 pmol dose, which was aimed at exploring an extremely high concentration of CgA, was completely inactive.

Recombinant and natural CgA (30 pmol, i.p.) exerted similar effects in the WEHI-164 and LLC models (Suppl. Figure S1). Furthermore, decreasing the dose to 6 pmol reduced the anti-tumor effects in both cases (not shown). Thus, natural CgA and recombinant CgA exerted similar anti-tumor effects in these models.

Optimal activity in all subcutaneous models was achieved with i.p. injection of 30 pmol of CgA (Figure 1A-1C), whereas i.v. injection of the same dose was less effective (data not shown). Pharmacokinetic analysis showed that the i.p. route generated circulating peak levels of about 3-4 nM CgA, whereas the i.v. route generated 17 nM levels (Suppl. Figure S2). This may explain the paradoxical lower activity of the i.v. *versus* the i.p. route. Accordingly, in the lung-colonization model, which was based on i.v. administration, maximal activity was obtained with a lower dose of recombinant CgA (4 pmol) (Figure 1D).

### The C-terminal region of CgA (residues 410-439) is crucial for its anti-tumor activity

**Figure 2:**
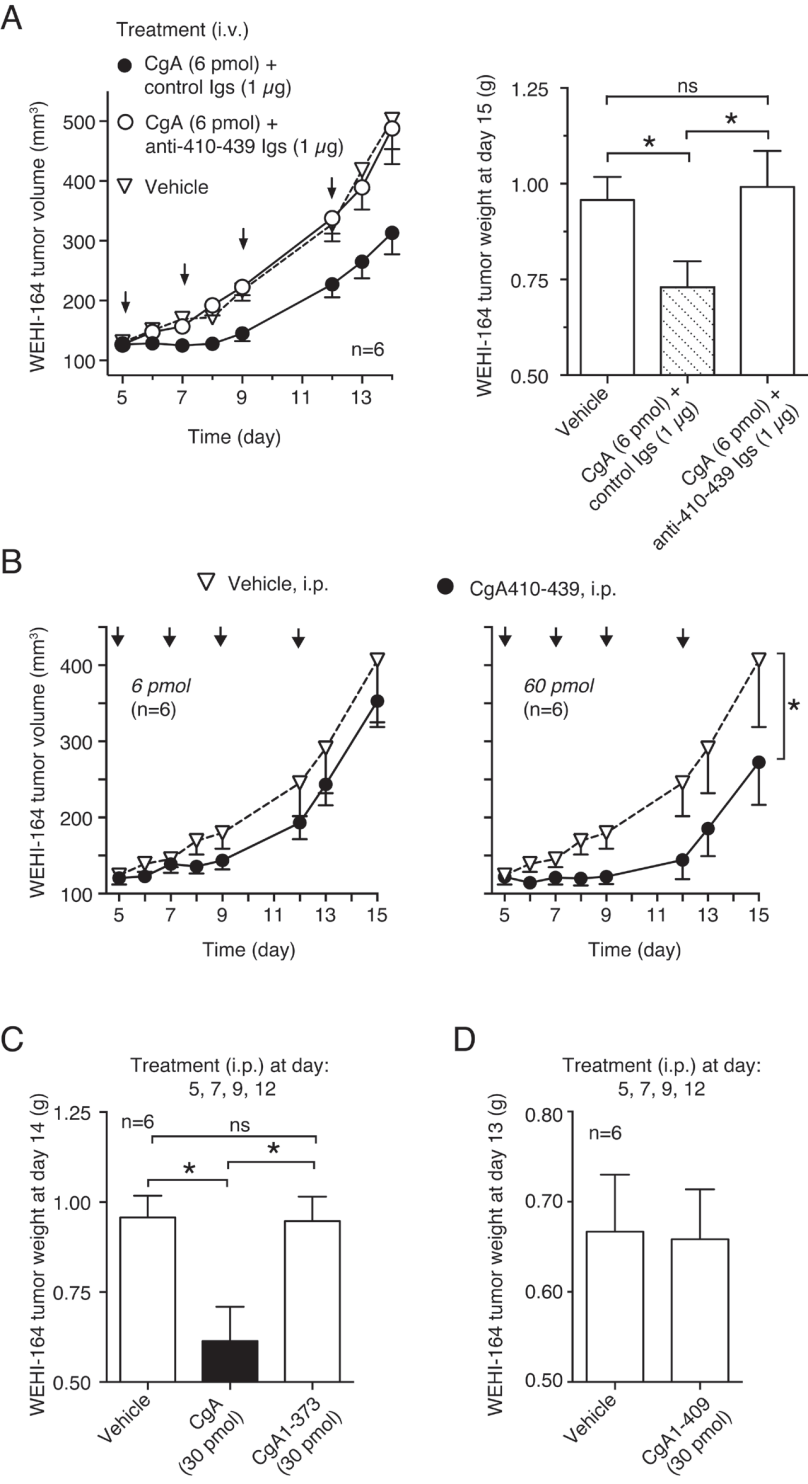
Role of CgA410-439 C-terminal region on the anti-tumor activity of CgA in the WEHI-164 model **A.** *Effect of anti-CgA antibodies on the anti-tumor activity of CgA*. Tumor-bearing mice were treated (i.v.) at the indicated time (arrows) with 6 pmol of recombinant CgA, alone and in combination with purified anti-CgA_410-439_ immunoglobulins (Igs). Tumor volumes and weights are shown (mean±SE, *n* = 6 mice/group). *, *P* < 0.05, by *t* test (2-tailed). **B.** *Anti-tumor activity of CgA peptide*. Mice were treated (i.p., arrows) with the indicated doses of peptide. Tumor volume (mean ± SE). The area under the curve (from day 5 to day 13) for each mouse was calculated using the GraphPad Prism Software. Statistical analysis was performed by *t* test (2-tailed) on the calculated areas (6 animals per group; *, *P* < 0.05). **C.***-***D.** *Anti-tumor activity of CgA, CgA and CgA*. Tumor bearing mice were treated at day 5, 7, 9, and 12 (i.p.) with 30 pmol of recombinant CgA or fragments lacking the C-terminal region CgA_1-409_and CgA_1-373_. Tumor weights are shown (mean±SE, *n* = 6 mice/group). *, *P* < 0.05, by *t* test (2-tailed).

We have previously shown that CgA contains an anti-angiogenic site in the C-terminal region 410-439 [24]. To assess whether the C-terminal region contributes to the anti-tumor activity of CgA we analyzed: a) the effect of antibodies against the region 410-439 on the anti-tumor activity of exogenous recombinant CgA, b) the anti-tumor activity of CgA_1-409_ and CgA_1-373_ (i.e. fragments lacking the C-terminal region), and c) the anti-tumor activity of CgA_410-439_ (a fragment corresponding to the C-terminal region). Anti-human CgA_410-439_ immunoglobulins (Igs), but not control Igs, could block the anti-tumor activity of exogenous CgA in the WEHI-164 model (Figure 2A). Furthermore, the C-terminal fragment CgA_410-439_, but not CgA_1-373_ and CgA_1-409_, could inhibit tumor growth (Figure 2B-2D). These findings suggest that the site responsible for the anti-tumor effects of full-length CgA is located in the C-terminal region.

### Neutralization of endogenous CgA with anti-CgA_410-439_ antibodies promotes tumor growth

**Figure 3:**
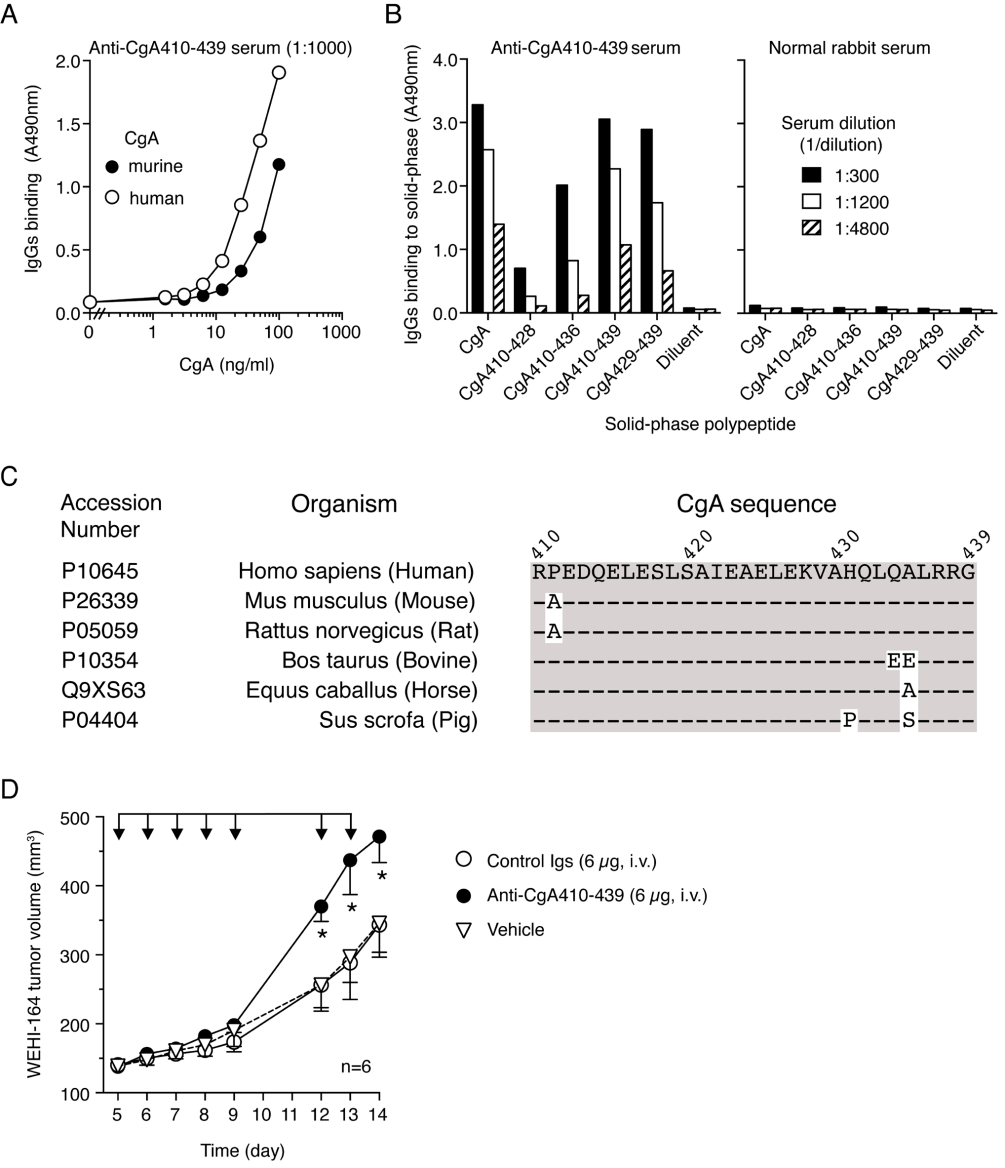
Neutralization of endogenous murine CgA with antibodies against the C-terminal region promotes the growth of subcutaneous WEHI-164 tumors **A.** *Cross-reactivity of anti-human CgA with human and murine CgA*. Human and murine CgA binding assay was performed by sandwich ELISA with mAb 5A8 (cross-reactive) in the capture step and with anti-human CgA_410-439_ in the detection step. **B.** *Epitope mapping of anti-CgA antibodies with synthetic peptides*. Binding of anti-CgA_410-439_ antibodies to various peptides corresponding to the C-terminal region of CgA. Antibody binding was detected with peroxidase-labeled secondary goat-anti-rabbit antibodies. Normal rabbit serum was used as negative control. **C.** *Alignment of CgA C-terminal sequence from the different species, showing high sequence conservation between human a murine CgA*. **D.** *Effect of anti-CgA antibodies on the growth of WEHI-164 fibrosarcomas in mice*. Tumor-bearing mice were treated (i.v.) with 6 μg of purified anti-CgA_410-439_ or control immunoglobulins (Igs) as indicated (arrows). Tumor volumes (mean±SE, *n* = 6 mice/group) are shown. *, *P* < 0.05, by Mann Whitney test.

To assess whether endogenous CgA, chronically produced and released in circulation by the neuroendocrine system, has a role in the regulation of tumor progression, we investigated the effect of CgA ablation on tumor growth in mice. Considering the crucial role of this protein in secretory granule biogenesis [30-34] and the consequent risk that genetic ablation of CgA might affect the release of several potentially relevant hormones, we decided to pursue an immunological approach based on systemic injection of antibodies against the C-terminal region of CgA (which are expected to neutralize extracellular CgA but not intra-granular CgA because of a different accessibility). Notably, the rabbit anti-human CgA_410-439_ Igs described above could efficiently recognize the CgA_429-439_ epitope of human and murine CgA (Figure 3A-3B), a sequence that is identical in these proteins (Figure 3C). Thus, this antibody was exploited to block the C-terminal region of endogenous circulating CgA in mice. Daily administration of this antibody (6 μg/mouse), but not normal rabbit Igs, significantly enhanced the growth rate of subcutaneous WEHI-164 tumors (Figure 3D). These results suggest that physiological levels of endogenous (natural) CgA can indeed reduce the growth rate of tumors.

### CgA inhibits angiogenesis in tumors with U-shaped dose-response curves

**Figure 4:**
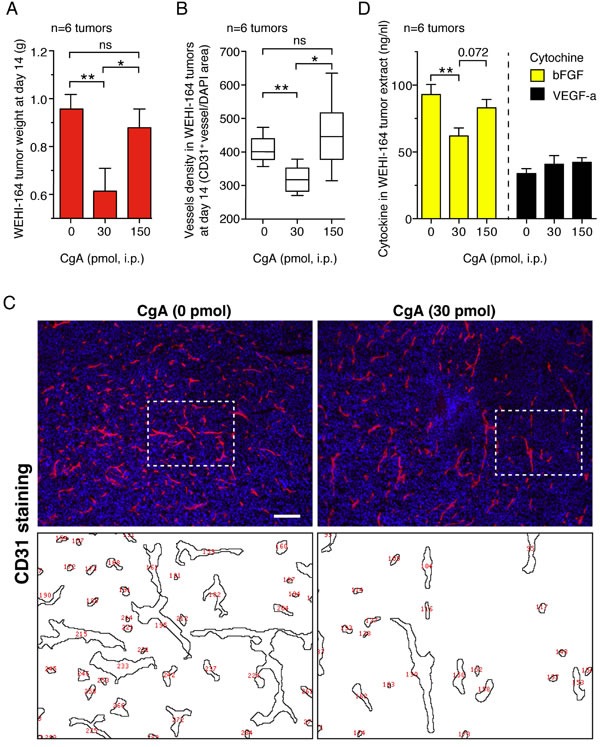
Effects of CgA on microvessel density and tumor growth in the murine WEHI-164 fibrosarcoma model **A.***-***C.** *Effects of CgA on tumor growth and vessel density*. BALB/c mice (*n* = 6/group) were treated, i.p., at day 5, 7, 9, and 11 after tumor implantation, with the indicated doses of recombinant CgA. Tumors were excised and weighted. Tissue sections were stained with anti-CD31 antibody (mAb MEC 13.3) and AlexaFluor 546 goat anti-rat IgG (red, endothelial staining) and with 4,6-diamidino-2-phenylindole (DAPI) (blu, nuclear staining). Vessel density was quantified by counting the number of red spots (CD31^+^) in each field analyzed by fluorescence microscopy (10 fields/section, 3 sections/tumor, 6 tumors/group, see “*Methods*”). Tumor weight (mean±SE) *(A)* and vessel density (box-plots with median, interquartile and 5-95 percentile values) **B.** at day 14 are shown. *, *P* < 0.05; **, *P* < 0.01, by two-tailed *t* test. Representative images of CD31 staining (10x magnification; bar, 200 μm) **C.** Images obtained after elaboration for vessel counting (red numbers, corresponding to area delimited by dashed lines) are also shown. **D.** *Effect of CgA on bFGF and VEGF-a expression in tumors*. bFGF in tumor tissue extracts was analyzed by ELISA.

The mechanism of the anti-tumor activity of full-length CgA was then investigated. No effect on tumor cell proliferation and viability was observed with *in vitro* cultures of tumor cells (data not shown), suggesting the *in vivo* effect of CgA on tumor growth was indirect, possibly related to stromal components. To assess whether CgA could regulate tumor angiogenesis, we investigated the effect of 30 and 150 pmol doses of recombinant CgA on tumor microvessel density by immunofluorescence microscopy in the WEHI-164 model. Staining of tumor tissue sections with an anti-CD31 antibody (a marker of endothelial cells) at day 14 showed that 30 pmol, but not 150 pmol of CgA, could significantly reduce vascular density (Figure 4A-4C). Interestingly, 30 pmol CgA, but not 150 pmol (i.p.), could reduce bFGF in tumor tissue extracts, a proangiogenic cytokine (Figure 4D). These data support the hypothesis that low-dose CgA can trigger anti-angiogenic mechanisms in tumors.

**Figure 5:**
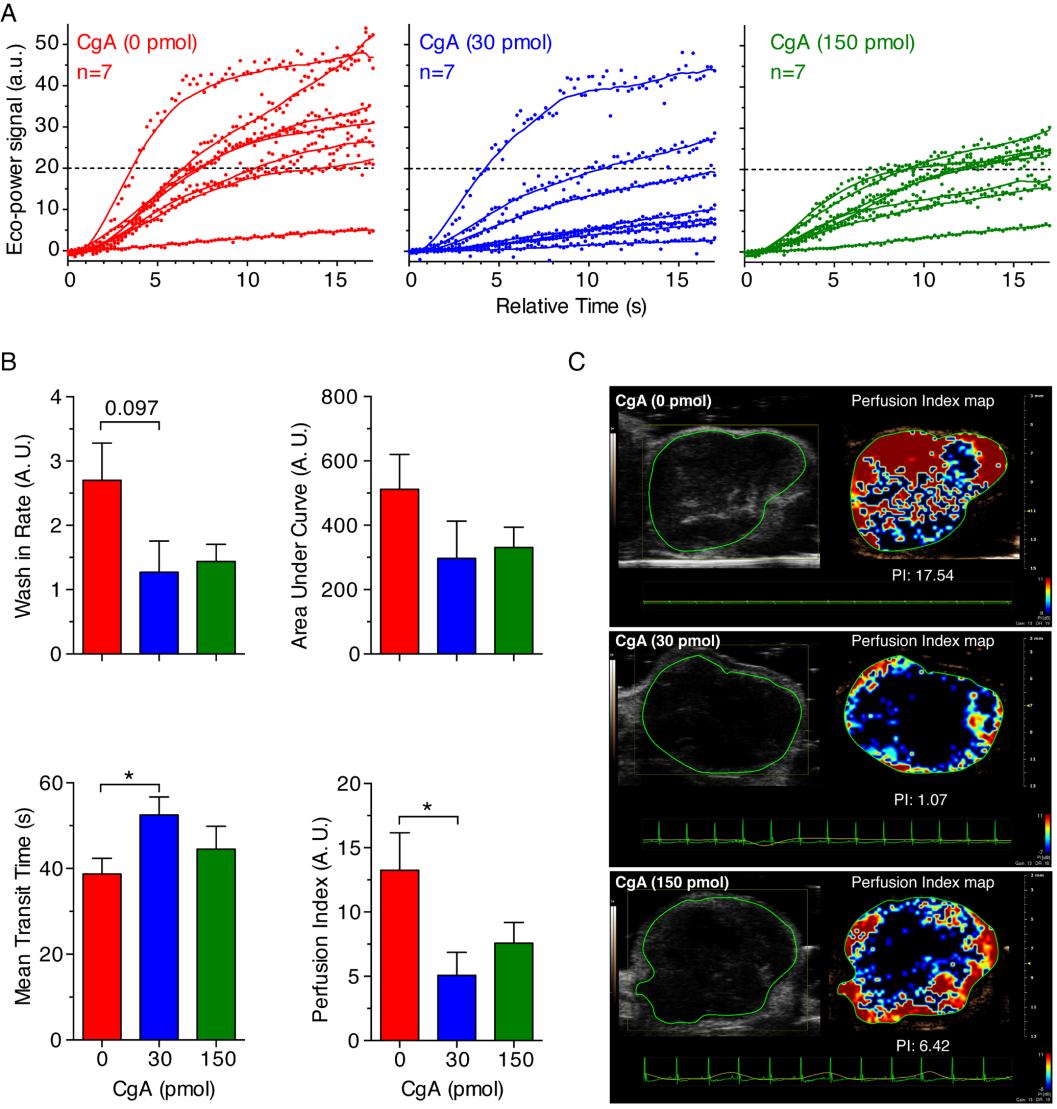
Effect of CgA on tumor perfusion as determined by Contrast Enhanced Ultrasound (CEUS) imaging WEHI-tumor-bearing mice (*n* = 7/group) were treated, i.p., with the indicated dose of recombinant CgA at day 6, 8, and 10 after tumor implantation and analyzed by CEUS imaging at day 15-16. MicroMarker Contrast Agent was injected at time 0 and its uptake was recorded for at least 17 s. **A.** *Wash-in curves of each mouse*. **B.** *Quantitative analysis of tumor perfusion*. Quantitative analysis was performed using VevoCQ^TM^ software. Perfusion parameters (Wash-in Rates, Area Under Curve, Mean Transit Time) were calculated using a region of interest (ROI, green lines in panel C) corresponding to the entire tumor area. Perfusion index corresponds to the ratio Area Under Curve/Mean Transit Time. Bars: mean ± SE; *, *P* < 0.05, by Mann-Whitney test. **C.** *Gray-scale tumor images (left) and color-coded Perfusion Index Maps of representative tumors treated with 0, 30 and 150 pmol of CgA (right)*. Red and blue areas correspond to high and low perfused tumor areas, respectively.

This view is further supported by the results of contrast enhanced ultrasound analysis of tumors (CEUS), performed on a different group of mice, showing that treatment with 30 pmol of CgA (i.p.), but not 150 pmol, could significantly reduce the tumor perfusion index (Figure 5A-5C).

The observed reduction of tumor weight, microvessel density, and perfusion by low-dose CgA suggests that regulation of angiogenesis is an important mechanism of action of its anti-tumor effects.

### CgA and CgA_410-439_ inhibit capillary structure formations from cultured aorta rings with U-shaped dose-response curves

**Figure 6:**
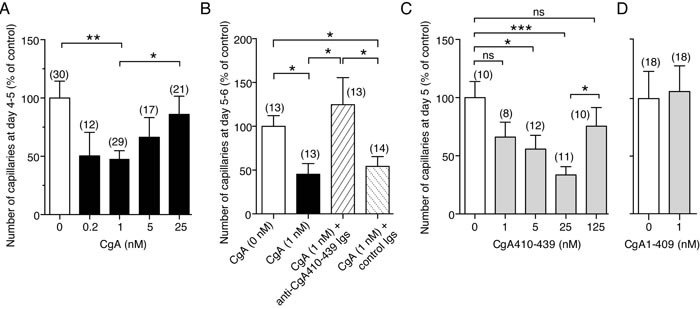
Effects of CgA and its C-terminal region on capillary sprouting from rat aortic rings (RAR) **A.***-***D.** *Effect of CgA, CgA CgA and anti-CgA antibodies in the RAR assay*. Bars represent the number of capillary-like structures emerging from the aorta rings treated as indicated, expressed as percentage of the untreated control (mean ± SE of 2-3 experiments). The number rings used (obtained from 2-4 rats) is indicated in each panel (n). *, *P* < 0.05; **, *P* < 0.01; ***, *P* < 0.001, by *t* test (2-tailed). RAR assays were performed as described previously [24, 43]. This method is based on the use of rat aorta rings cultured in three-dimensional collagen gels and on the measurement of the number of capillary-like structures sprouting from rat aorta rings after 4-5 days. Basal angiogenesis, obtained without addition of bFGF or VEGF, was examined in the absence or presence of various doses of recombinant CgA or CgA_410-439_. Anti-CgA_410-349_ and control Igs (20 μg/ml) were also added as indicated.

To provide further information on the mechanisms underlying the inhibition of angiogenesis by CgA in tumors, we evaluated the effect of various doses of CgA, CgA fragments and anti-CgA Igs on the spontaneous sprouting of capillary-like structures from rat aortic rings (RARs) cultured in collagen gels. Full-length CgA could exert significant inhibitory effects in this assay with a U-shaped dose-response curve, with the maximal activity being obtained with 0.2-1 nM recombinant CgA (Figure 6A). Notably, the anti-CgA_410-439_ Igs neutralized the effect of CgA (Figure 6B), as observed in the *in vivo* experiments. Furthermore, the peptide CgA_410-439_ recapitulated, albeit at higher doses, the U-shaped inhibitory effects of CgA (Figure 6C). Finally, no anti-angiogenic effects were observed with 1 nM CgA_1-409_ (Figure 6D). These data support the hypothesis that the C-terminal region of CgA contains the structural determinants of the anti-angiogenic activity and suggest that cleavage of this region causes a marked loss of anti-angiogenic potency, CgA_410-439_ being markedly less active than CgA_1-439_.

### Endothelial cells are an important target of CgA and CgA_410-439_

**Figure 7:**
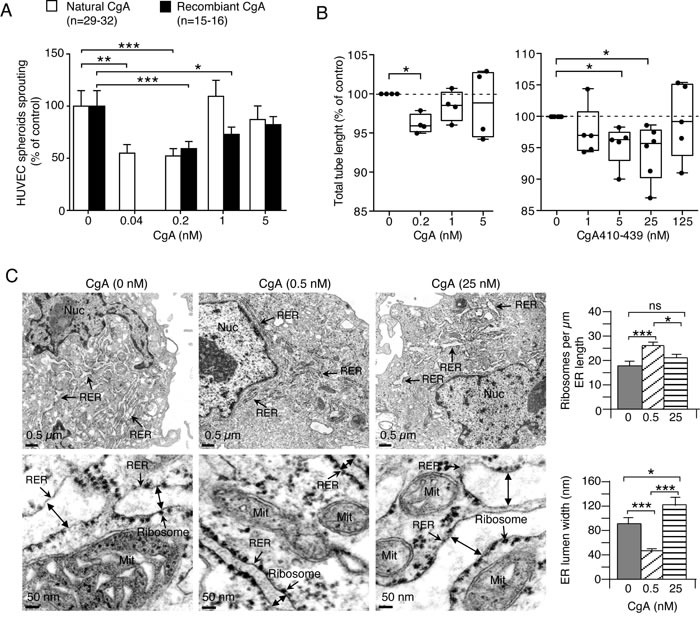
Effects of CgA and its C-terminal fragment on endothelial cells **A.** *Effect of CgA on endothelial spheroid sprouts formation*. HUVEC spheroids were cultured in 3D-collagen gel in the presence of various concentrations of natural or recombinant CgA. After overnight incubation, sprouts were counted using an inverted-phase contrast microscope. Cumulative results of 2-3 experiments (8-16 spheroids/experiment). ***, *P* < 0.001; **, *P* < 0.01, *, *P* < 0.05, by *t* test. **B.** *Effect of CgA and CgA on tube formation by cultured HUVECs*. Cumulative results of 4-6 experiments (6 replicates/experiment). *, *P* < 0.05, by *t*-test (one-sample). Endothelial tube assays were performed with HUVECs and μ-Slide Angiogenesis ibiTreat plates (Ibidi) coated with 10 μl of reduced-growth factor Basement Membrane Extract (BME) (Cultrex). HUVECs (2 × 10^4^ cells in EGM-2 medium, 50 μl/well) were seeded in the presence or absence of various doses of recombinant CgA or CgA_410-439_. After overnight incubation, the 2-dimensional tube organization was examined using an inverted-phase contrast microscope (LEICA, BM, IRB equipped with 10X objective lens and AxioVision acquisition software) and photographed. Photographs were then analyzed by ImageJ software using the Angiogenesis Analyzer tool (http://image.bio.methods.free.fr/ImageJ/?Angiogenesis-Analyzer-for-ImageJ.html&lang = en). **C.** *Effect of CgA on the subcellular morphology of endothelial cells*. TEM images of HUVECs after treatment with 0, 0.5, and 25 nM recombinant CgA for 12 h as indicated. Double-head arrows, ER lumen width; Mit, mitochondria; Nuc, nucleus; RER, rough endoplasmic reticulum. Histograms show quantitative descriptions of ER-bound ribosomes and ER-lumen size, as determined using NIH ImageJ software on 30 images from 15 cells (two images/cells) *(right)*. *, *P* < 0.05: ***, *P* < 0.001, by *t* test (2-tailed).

To identify the cellular targets of the anti-tumor activity of CgA we then investigated its effects on cultured endothelial cells. Both natural and recombinant CgA could inhibit the sprouting of capillary-like structures from HUVEC spheroids cultured in three-dimensional collagen gels with U-shaped dose-response curves and similar potency (maximal activity: 0.04-0.2 nM CgA) (Figure 7A). Recombinant CgA and synthetic CgA_410-439_ exerted modest, albeit significant, inhibitory effects on the formation of capillary-like structures in a two-dimensional endothelial tube formation assay, again with U-shaped dose-response curves (Figure 7B). These data suggest that CgA can directly affect the physiology of endothelial cells. No effect on endothelial cell proliferation and viability was observed (not shown). Of note, the inhibitory effects observed in the tube formation assay were lower than those observed with RAR and HUVEC spheroid assays, suggesting that inhibition of three-dimensional matrix invasion is an important mechanism.

### CgA regulates endoplasmic-reticulum (ER) lumen width and number of ER-bound ribosomes in endothelial cells with bimodal-dose response curves

The effects of CgA at low and high concentrations on endothelial subcellular morphology were then analyzed by transmission electron microscopy. Low-dose recombinant CgA (0.5 nM) increased the number of ER-bound ribosomes/μm of ER length (by 44%), whereas high-dose CgA (25 nM) had no effect (Figure 7C). Furthermore, low-dose CgA reduced ER-lumen width (by 50%), as compared to saline-treated group, whereas high-dose CgA increased the lumen width (Figure 7C). Although the relationship between these changes in subcellular morphology and angiogenesis remains to be established, these results support the hypothesis that CgA can directly affect the physiology of endothelial cells with U-shaped dose-response curves.

### CgA induces protease nexin-1 mRNA in endothelial cells with a bell-shaped dose response curve

**Figure 8:**
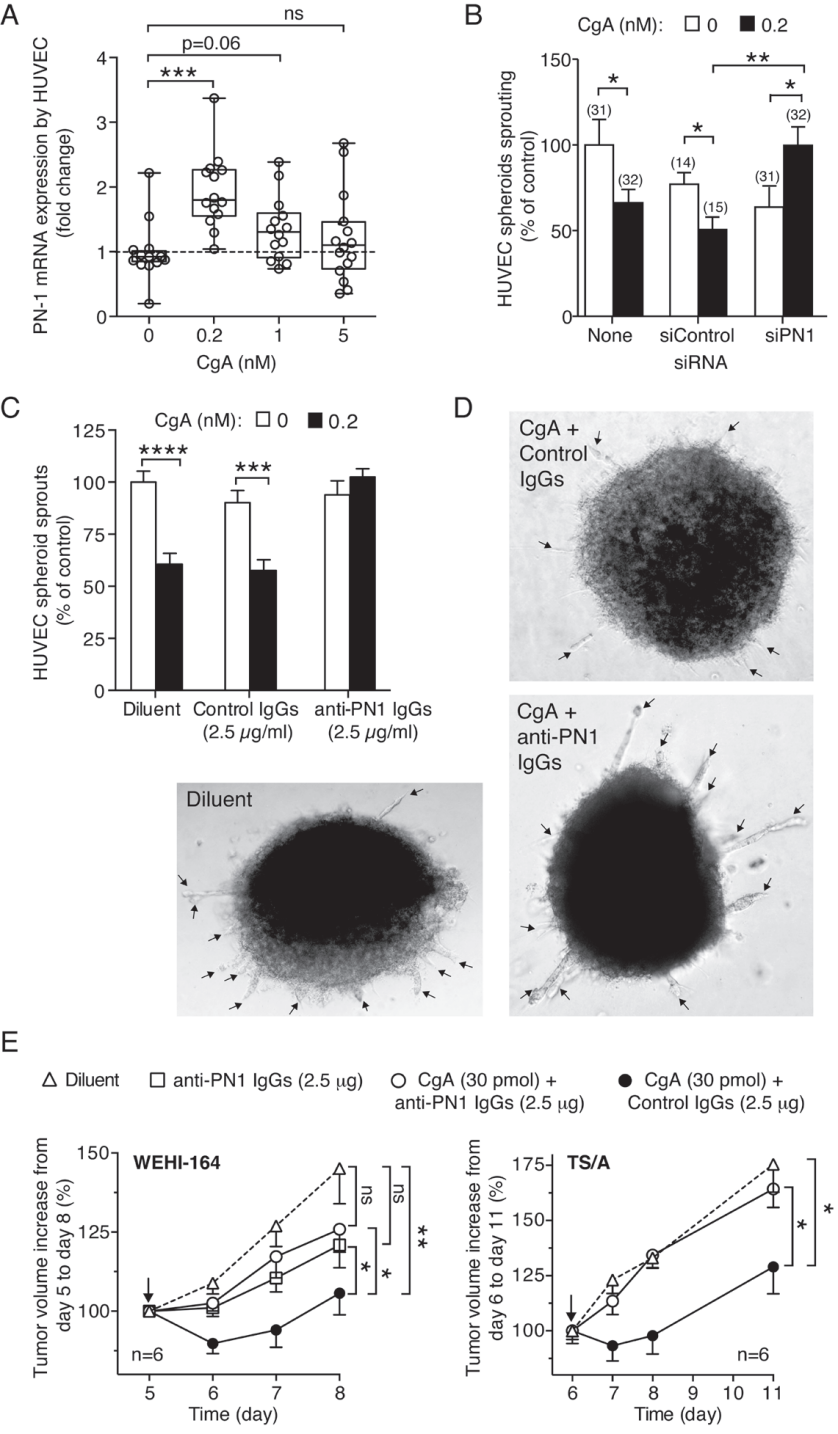
Role of PN-1 on CgA anti-tumor activity **A.** *Induction of protease nexin-1 mRNA in endothelial cells*. HUVECs were treated with various doses of recombinant CgA for 20 h. The presence of PN-1 transcripts in cells was analyzed by real time PCR. PN-1 mRNA expression is reported as fold change compared to untreated cells. The cumulative results of 3 experiments are shown: in each experiment each sample was analyzed 5-6 times by real-time PCR (each in triplicate). Box-plots with median, interquartiles and 5-95 percentile values are shown (dotted line, mean value of controls). ***, *P* < 0.001, by *t* test (2-tailed). **B.** *Effect of PN1 gene silencing on the activity of CgA in the endothelial spheroid sprouting assay*. HUVEC cells spheroids were transfected with PN-1 siRNA mixture (siPN1) or with control siRNA (siCtrl) as described in Methods. After transfection, spheroids were treated with or without and 0.2 nM recombinant CgA_1-439_ for 16 hours. The number of sprouts was from each spheroid was then counted. Cumulative results of two independent experiments (8-16 spheroids/experiment). The number of spheroids used is indicated in each panel (*n*). *, *P* < 0.05; **, *P* < 0.01 by *t* test. **C.***-***D.** *Effect of anti-PN-1 antibodies on the activity of CgA in the endothelial spheroid sprouting assay*. Cumulative results of two independent experiments (8 spheroids/experiment, total *n* = 16). Spheroids were incubated with or without recombinant CgA (0.2 nM) in the absence or the presence of anti-PN-1 polyclonal immunoglobulins (goat IgGs anti-human Serpin E2/PN1), or control immunoglobulins (normal goat IgGs) as indicated. Bars: mean ± SE. ****, *P* < 0.0001, ***, *P* < 0.001, by *t* test (2-tailed). Representative microphotographs of spheroids are shown in (*C*) (arrows: endothelial cell sprouts). **E.** *Effect of anti-PN-1 antibodies on the anti-tumor activity of CgA*. WEHI-164- or TS/A-tumor-bearing mice were treated with recombinant CgA (i.p.) in combination with anti-PN-1, or with control immunoglobulins (normal goat IgGs) at the indicated doses. Antibodies were given 2.5 h before CgA. Arrows indicate the day of treatment. Tumor volume (mean ± SE). The area under the curve for each mouse was calculated using the GraphPad Prism Software. Statistical analysis was performed by Mann-Whitney test on the calculated areas (6 animals per group; **, *P* < 0.01; *, *P* < 0.05).

To investigate the mechanism of the anti-angiogenic activity of CgA we then analyzed the effect of CgA on the endothelial expression of protease nexin-1 (PN-1), a serine protease inhibitor (serpin). The rationale for this study relies on the notion that a peptide corresponding to CgA_411-436_ can induce, at relatively high concentrations, PN-1 in AtT-20 pituitary cells [35], and that this protein is an important anti-angiogenic factor [36]. Interestingly, recombinant CgA induced the production of PN-1 mRNA in endothelial cells with a bell-shaped dose response curve, with the PN-1 mRNA expression being obtained after stimulation with 0.2 nM CgA, but not with 5 nM CgA (Figure 8A). This finding further support the concept that endothelial cells are important targets of CgA and suggest that PN-1 induction could be an important mechanism of its anti-tumor activity.

### PN-1 gene silencing and anti-PN-1 antibodies inhibit the anti-angiogenic and anti-tumor activity of CgA

To assess whether the anti-angiogenic and anti-tumor activity of CgA was indeed mediated by PN-1, we investigated a) the effect of siRNA-mediated PN-1 gene silencing and of anti-PN-1 neutralizing antibodies on the anti-angiogenic activity of CgA in the endothelial-sprouting assay (spheroids), and b) the effect of anti-PN-1 neutralizing antibodies on the anti-tumor activity of CgA in the WEHI-164-fibrosarcoma and TS/A adenocarcinoma models, *in vivo*. PN-1-siRNA and anti-PN-1 neutralizing antibodies, but not control siRNA and antibodies, completely abolished the anti-angiogenic effect of 0.2 nM recombinant CgA (Figure 8B-8D). Furthermore, the anti-tumor effects of 30 pmol CgA were significantly inhibited by anti-PN1 antibodies, but not by control antibodies in both fibrosarcoma and adenocarcinoma models (Figure 8E). These results support the hypothesis that PN-1-induction by CgA is a crucial mechanism of its anti-tumor activity.

## DISCUSSION

The results show that full-length CgA, a protein released in circulation by the neuroendocrine system, can regulate the growth of solid tumors. This view is supported by the results of *in vivo* studies performed in different murine models of non-neuroendocrine tumors, including fibrosarcoma, mammary adenocarcinoma, lung carcinoma, and primary and metastatic melanoma. In particular, the results obtained in the fibrosarcoma model show that neutralization of endogenous CgA with specific antibodies enhances tumor growth rate, whereas systemic administration of low amounts of exogenous full-length CgA reduces tumor growth. It appears, therefore, that circulating full-length CgA works as an inhibitor that delays tumor growth. Notably, dose-escalation studies showed that CgA affects tumor growth with a U-shaped dose-response curve in all tumor models studied, with the maximal inhibitory activity being obtained with biweekly injections of 30 pmol of CgA (i.p.). This dose, administered at 2-3 day intervals, generates peak plasma levels of about 3-4 nM that progressively declines to 0.5-1 nM in 7-8 h and to even lower concentrations at later time points. Considering that circulating levels of CgA in normal subject is 0.2-1 nM (most consisting of fragments lacking the C-terminal region and less than 10-20% consisting of full-length CgA) [25], the biological effects observed in our models are likely patho-physiologically relevant.

What is the mechanism of the anti-tumor activity of full-length CgA? The lack of effects on tumor cell proliferation and viability *in vitro* argues against the hypothesis that neoplastic cells are primary targets of CgA in our models. More likely, stromal components of the neoplastic tissues are affected. According to this view, the results of *in vivo* mechanistic studies (by immunohistochemistry and contrast enhanced ultrasound analysis) show that CgA can reduce microvessel density and tumor perfusion, suggesting that CgA targets the tumor vasculature. Notably, the U-shaped inhibitory effects on angiogenesis mirror those on tumor mass, supporting the hypothesis that reduction of angiogenesis is indeed an important mechanism of the anti-tumor activity of CgA. This view is also supported by the results of *in vitro* angiogenesis assays, showing that CgA inhibits capillary sprouting from rat aortic rings and HUVEC spheroids with U-shaped dose-response curve, maximal activity being obtained with 0.1-0.2 nM CgA (i.e. with physiologically relevant concentrations).

The results of studies aimed at elucidating the structure-function relationships of CgA show that the C-terminal region (residues 410-439) contains the active site. Notably, antibodies against this region, which is highly conserved in mice, humans and other species (see Figure 3C), could neutralize the anti-tumor activity of both endogenous and exogenous CgA. Furthermore, a synthetic peptide corresponding to this sequence could inhibit tumor growth and recapitulate the U-shaped inhibitory effects induced by recombinant full-length CgA in the *in vitro* angiogenesis assay. However, it is noteworthy that maximal activity of CgA_410-439_ in the RAR assay was achieved with 5-25 nM, i.e. with 25-fold higher concentration compared to full-length CgA. Possibly, the conformational structure of this peptide is less stable than that of the cognate sequence of full-length CgA. This finding may have important biological implications, as proteolytic cleavage of CgA C-terminal region might cause a marked loss of anti-angiogenic potency. These data also imply that the production of factors capable of promoting or inhibiting CgA cleavage in tumors, such as specific proteases or protease inhibitors, may be relevant for the anti-angiogenic activity of circulating CgA. Regarding C-terminal fragmentation of CgA it might be relevant also to note that the human and murine CgA sequence contains a proline or an alanine, respectively, at position 411, which may cause differential susceptibility to proteases and, consequently, generation of C-terminal peptides of different length and conformational stability.

What are the cellular targets of the CgA anti-angiogenic activity? The finding that CgA can inhibit endothelial cell sprouting and capillary tube formation with U-shaped dose-response curves suggests that the endothelium is an important target of CgA. Regulation of its physiology might be, therefore an important mechanism of its anti-tumor activity. Notably, in these cells CgA regulated, with U-shaped dose-response curves, the number of ER-bound ribosomes and the ER-lumen size. Although the relationships between these morphological changes with angiogenesis remains to be elucidated, these findings support the concept that CgA can directly affect the physiology of endothelial cells. This hypothesis is further supported by the observation that CgA can also induce in endothelial cells the protease nexin-1 (PN-1) mRNA transcript, a serine protease inhibitor (serpin), with a bell-shaped dose-response curve (maximal activity, 0.2 nM). Remarkably, recent studies have shown that PN-1 inhibits endothelial cell migration, capillary tube formation and angiogenesis in various *in vitro* and *in vivo* assays, independently of its serpin activity [36], and that overexpression of PN-1 inhibits angiogenesis and prostate adenocarcinomas growth in mice [37]. Thus, the induction of PN-1 by CgA may contribute to its anti-angiogenic and anti-tumor activities. This view is supported by our observation that anti-PN1 antibodies could neutralize the anti-angiogenic activity of CgA *in vitro* as well as the anti-tumor activity of CgA in different *in vivo* models. Moreover, considering that CgA is a good substrate of thrombin and plasmin [24, 25], often present in tumors, and that PN-1 is a potent inhibitor of these proteases [38], it is tempting to speculate that PN-1 may also serve to prevent the local cleavage of CgA in tumors (thereby preserving its anti-angiogenic activity) and/or other factors necessary for tumor progression and invasion. Finally, the bell-shaped dose-response curve and the consequent lack of PN1 induction by high concentrations of CgA may explain the lack of anti-tumor effects with high-dose CgA.

In conclusion, the results suggest that circulating full-length CgA can impair angiogenesis and tumor growth, and that cleavage of its C-terminal region markedly reduces its activity. Although CgA is a widely studied histopathological and serological marker for neuroendocrine tumors, this is the first evidence that circulating full-length CgA, released in the blood by the neuroendocrine system, has a functional role in non-neuroendocrine tumors. This may have important pathophysiological, prognostic and therapeutic implications. First, a decrease of CgA blood levels and/or an increase of its fragmentation might promote disease progression. Thus, pharmacological treatments aimed at restoring the balance of pro-/anti-angiogenic forms of CgA in patients might represent novel therapeutic strategies. Second, detection of full-length CgA levels and its fragmentation might have a diagnostic/prognostic value. At this regard, we have recently shown that fragmentation of the CgA C-terminal region in the bone marrow of multiple myeloma patients is associated with increased micro-vessel density, a known marker of disease activity [25], and is a prognostic indicator of poor outcome in patients with pancreatic adenocarcinoma (manuscript in preparation).

Finally, it is important to say that the observations reported here, obtained with non-neuroendocrine tumor models, cannot be extrapolated to neuroendocrine tumors, as neuroendocrine tumor-derived CgA might reach local concentrations out of the range studied here.

## MATERIALS AND METHODS

### Cell lines and reagents

Human umbilical vein endothelial cells (HUVECs) were from Lonza, Walkersville, MD) and cultured as recommended by the supplier. Murine WEHI-164 fibrosarcoma, B16-F1 and B16-F10 melanoma, and TS/A mammary adenocarcinoma cells were cultured in DMEM or RPMI with standard supplements. Recombinant human full-length CgA_1-439_, CgA_1-409,_ CgA_1-373,_ and synthetic CgA_410-439_ were prepared as described [24]. Natural human CgA was purified fron pheochromocytoma tissue extract as described [39]. Monoclonal antibodies (mAbs) B4E11 (epitope: CgA_68-71_) and 5A8 (epitope: CgA_53-57_) were prepared as described previously [40, 41]. Anti-human CgA and anti-CgA_410-439_, were raised in rabbits by immunization with human recombinant CgA_1-439_, or with a synthetic peptide encompassing the sequence CgA_410-439_ coupled to keyhole limpet hemocyanin (Primm, Italy). Rabbit anti-CgA_410-439_ immunoglobulins (Igs) were purified by affinity chromatography using the CgA_410-439_ peptide coupled to Activated CH-Sepharose (GE, Amersham). Goat IgGs anti-mouse serpin E2/PN1, goat IgGs anti-human serpin E2/PN1 and normal goat immunoglobulins were from R&D System.

### ELISA

Endogenous CgA present in circulation in mice was detected by sandwich ELISA based on the use of mAb 5A8 (cross-reactive with murine CgA) and a rabbit polyclonal antibody against recombinant murine CgA in the capturing and detection steps, respectively. Exogenous human CgA in murine plasma samples was detected by a similar sandwich ELISA based on the use of mAb B4E11 (specific for human CgA) and rabbit polyclonal antibodies against human CgA, as described [12].

### *In vivo* studies

Studies in animal models were approved by the Ethical Committee of the San Raffaele Scientific Institute and done according to the prescribed guidelines.

BALB/c and C57BL/6/N female mice (6-7 weeks old, from Charles River Laboratories, Calco, Italy) were challenged with s.c. injection in the left flank of 2×10^5^ B16-F1 melanoma cells (C57BL/6/N), 2×10^5^ TS/A cells (BALB/c), or 1.5×10^6^ WEHI-164 cells (BALB/c). Mice were treated intra-peritoneally (i.p.) with full-length CgA_1-439,_ CgA_1-409,_ CgA_1-373_ or CgA_410-439_ in 0.9% sodium chloride containing 100 μg/ml endotoxin-free human serum albumin. Drug doses and treatment schedules for each experiment are reported in figures and figure legends. Tumor growth was monitored daily by measuring the tumor size with calipers [24].

### Contrast enhanced ultrasound analysis of tumors

Contrast enhanced ultrasound (CEUS) was performed on BALB/c mice treated at day 6, 8, and 10 after tumor implantation, with 0, 30 and 150 pmol of CgA (i.p.), respectively. Analysis was performed using a high-performance ultrasonographic scanner designed for small animal imaging (Vevo 2100; Visual Sonics, Toronto, Canada). Mice were anesthetized with isoflurane (flurane; Isoba, Schering-Plough, USA, 4% in oxygen for induction, 2% for maintenance at a rate of 1 l/min). The tail vein was catheterized before imaging using a 27-gauge and one-half inch butterfly catheter. Mice were positioned in a supine position. ECG and respiration rate were monitored during the examination. CEUS studies were performed in contrast mode using the MS250 linear transducer (fc = 21 MHz, 13-24 MHz; Vevo 2100) during intravenous bolus injection of Vevo MicroMarker untargeted ultrasound contrast agent (CA) (Bracco, Geneva, Switzerland; VisualSonics), prepared in agreement with the specifications of the producer. A total volume of 50 μl of microbubble suspension (4.3×10^7^ microbubbles/bolus) was injected in 4 seconds. Sonographic data acquisition started immediately after CA injection and 17 s cine loops of contrast wash-in were acquired. Imaging parameters were held constant throughout each experiment to reduce variability (transmit power 4%; dynamic range 40 dB; center frequency 18 MHz; frame rate 20 Hz; contrast gain 53 dB; gate 4; beam-width standard). Recorded cine loops were processed for time-intensity curve (TIC) analysis using VevoCQ™ Advanced Contrast Quantification Software (Visual Sonics). A region of interest (ROI) was drawn along the perimeter of each tumor. The time-intensity curve for each imaging protocol was plotted and a mathematical equation model based on log-normal distribution function was used to fit the contrast uptake time-intensity curve [42]. Perfusion parameters extracted from the fitted model were the Area Under the Curve (AUC), the wash-in rate (WiR) defined as the maximum slope of the fitted curve, the mean transit time (mTT) defined as the average time required for the contrast agent to pass through the ROI and perfusion index (PI) defined as AUC divided by mTT.

### Tumor vasculature density analysis

Tumor vessel density was estimated by CD31-immunofluorescence staining of tumor tissue sections (7 μm, prepared by cutting tumors at three different levels) as previously described [24]. Each section was analyzed using an Axioplan2 fluorescence microscope (Zeiss, equipped with 10X objective lens and AxioVision acquisition software). Images of tissue sections (10 fields/section, 3 sections/tumor, 6 tumors/group) were randomly acquired. Vessel density was quantified by counting the number of red spots (CD31^+^) in each image using the ImageJ software (NIH, USA) as follows: each image was thresholded to highlight structures corresponding to CD31^+^ vessels; the resulting image was processed with the command “Binary” and “Close-” to link adjacent structures. The number of the resulting structures was then electronically counted and normalized on DAPI staining.

### Real-time PCR

HUVECs were seeded in 12-well microtiter-plates. Six hours later, the cells were incubated for 20 h with various amounts of full-length CgA in cell culture medium consisting of 30% EGM-2 and 70% EBM-2 mixture. Total RNA was isolated from HUVECs using RNeasy Mini Kit and reverse transcribed with QuantiTect Reverse Transcription Kit (Qiagen, Valencia, CA). Real-time PCR was performed, in triplicate on each sample, using the 7900HT Fast Real-Time PCR System and SYBR^®^ Green PCR Master Mix (Applied Biosystems^®^, Foster City, CA) according to the manufacturer's instructions. Specific primer sequences were as follows: PN-1-forward 5′-CCGCTGTCTGCCATCATCC-3′, PN-1-reverse 5′-AGAACTTTCAGCGGCTCCTT-3′. Glyceraldehyde-3-phosphate dehydrogenase (GAPDH) was used as a reference gene employing the following primers: GAPDH-forward 5′-AACGGATTTGGTCGTATTG-3′, GAPDH-reverse 5′-GGAAGATGGTGATGGGATT-3′. Relative mRNA expression levels were calculated using the 2^−ΔCt^ method.

### Transmission electron microscopy (TEM)

HUVECs were fixed with Trump's fixative (a mixture of 4% paraformaldehyde and 1% glutaraldehyde in 0.1 M cacodylate buffer) and post-fixed in 1% OsO_4_ in 0.1 M cacodylate buffer at 4°C. This was followed by staining with 2% uranyl acetate at 4°C, serial ethanol dehydrating, and lastly embedding as monolayers in Durcupan. Sections (50 to 60 nm) were cut using a Leica UCT ultra-microtome, and collected on 300 mesh grids. Grids were viewed using a JEOL 1200EX II (JEOL, Peabody, MA) TEM and photographed using a Gatan digital camera (Gatan, Pleasanton, CA). ER lumen width, length of the ER membrane on both sides of the ER lumen and the number of ER-bound ribosomes/μm of ER membrane were determined using NIH ImageJ software (30 images from 15 cells).

### Endothelial spheroid sprouting assay

To generate multicellular spheroids, HUVEC were resuspended in EBM-2 medium containing 5% FBS and 5% (w/v) carboxymethylcellulose (Sigma Aldrich,), seeded in a round-bottom Corning Spheroid 96-well microplate (4×10^4^ cells/well, 30 μl), and incubated overnight at 37°C, 5% CO_2_. Endothelial cell spheroids and their supernatant were transferred using a multichannel pipette into a 96-well flat-bottom microtiter plate (Corning™ 3631) containing CgA solutions (10 μl/well) in EBM-2 supplemented with 5% FBS. The spheroids were then mixed with a collagen solution (40 μl/well) consisting of EBM-2 supplemented with 0.5% carboxymethylcellulose, 10% Medium 199 (10x), 0.02 N sodium hydroxide, 5% of FBS, 0.2% of collagen-R (Serva). Spheroids were then incubated overnight at 37°C, 5% CO_2_, to allow sprout formation. Sprouts were counted using a ZEISS Axio Observer.Z1 microscope (10x magnification).

### PN-1 gene silencing in HUVEC spheroids

Human PN1(rGrGrArAr CrArGrArCrUrCrGrArUrGrCrArArGrUrGrUrUTC andrUrGrUrArC rUrGrArGrGrArArUrGrArArUrArGrArArArGGC) (siPN1) and scrambled (rCrGrUrUr ArArUrCrGrCrGrUrArUrArArUrArCrGrCrGrUAT) (siCtrl) siRNAs were synthesized by Origene (Rockville, MD). Paired siRNAs were first heated to 94°C for 2 min and then cooled to room temperature for annealing. HUVEC spheroids were mixed with 20 ml of Opti-MEM containing 20 nM siRNA, 0.35 ml lipofectamine RNAiMAX transfection reagent (Invitrogen) and incubated for 6 h (final volume of 50 ml). PN1 gene silencing was confirmed by PCR using the PN1 and housekeeping GAPDH (control) primers described above.

## SUPPLEMENTARY MATERIALS FIGURES


