# Supplementary material for: Regulation of tumor growth by circulating full-length chromogranin A
Source: Oncotarget. 2016 Sep 24;7(45):72716–32. doi: 10.18632/oncotarget.12237 (PMC5341939; doi:10.18632/oncotarget.12237)
Supplement: Supplementary file 1 [file oncotarget-07-72716-s001.pdf]

Regulation of tumor growth by circulating full-length chromogranin A

Supplementary Material

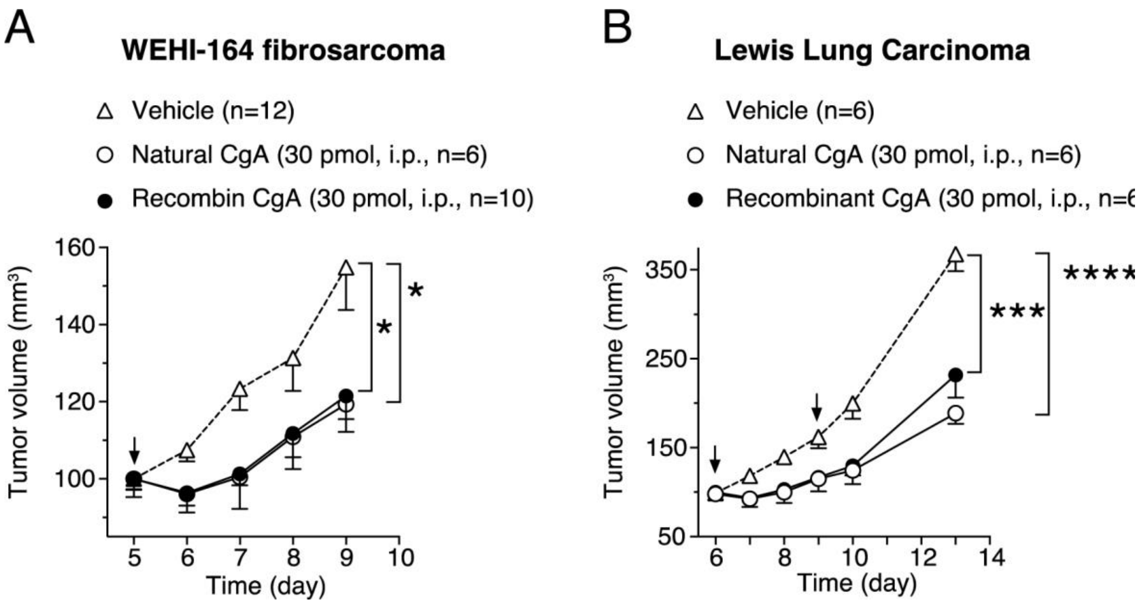

Figure S1. Effects of natural and recombinant CgA on tumor growth in the WEHI-164 fibrosarcoma and Lewis lung carcinoma models.

Tumor-bearing mice were treated (i.p.) at the indicated time (*arrows*) after tumor implantation with 30 pmol of natural or recombinant CgA. Tumor volumes are shown (mean±SE). The area under the curve for each mouse was calculated using the GraphPad Prism Software. Statistical analysis was performed by *one-way ANOVA test* on the calculated areas (*n*, mice/group; *, P < 0.05; ***, P < 0.001; ****, < 0.0001).

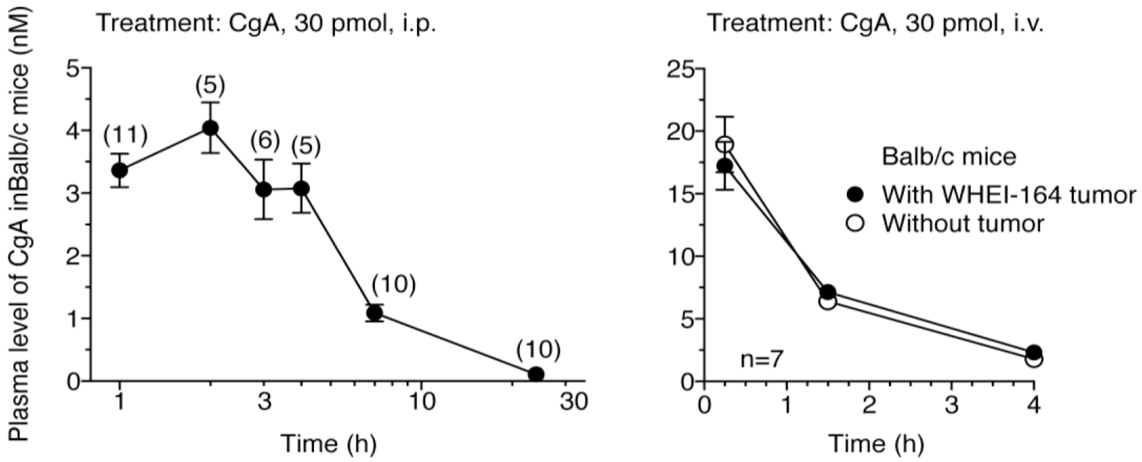

Figure S2. Plasma levels of human CgA in mice after intraperitoneal (i.p.) or intravenous (i.v.) injection of CgA.

Balb/c mice with or without subcutaneous WEHI-164 fibrosarcomas were injected with 30 pmol of CgA i.p. (*left*) or i.v. (*right*) at day 21 or 22 after tumor implantation. Blood was collected at various time points; plasma levels of CgA were measured by ELISA (mean±SE, n=5-11 mice/group).
